# Nanotechnology Formulations for Antibacterial Free Fatty Acids and Monoglycerides

**DOI:** 10.3390/molecules21030305

**Published:** 2016-03-03

**Authors:** Joshua A. Jackman, Bo Kyeong Yoon, Danlin Li, Nam-Joon Cho

**Affiliations:** School of Materials Science and Engineering and Centre for Biomimetic Sensor Science, Nanyang Technological University, 50 Nanyang Drive, Singapore 637553, Singapore; jjackman@ntu.edu.sg (J.A.J.); bokyeong001@e.ntu.edu.sg (B.K.Y.); lidanlin0319@gmail.com (D.L.)

**Keywords:** antimicrobial lipid, fatty acid, monoglyceride, nanotechnology, emulsion, liposome, solid lipid nanoparticle, hydrogel

## Abstract

Free fatty acids and monoglycerides have long been known to possess broad-spectrum antibacterial activity that is based on lytic behavior against bacterial cell membranes. Considering the growing challenges of drug-resistant bacteria and the need for new classes of antibiotics, the wide prevalence, affordable cost, and broad spectrum of fatty acids and monoglycerides make them attractive agents to develop for healthcare and biotechnology applications. The aim of this review is to provide a brief introduction to the history of antimicrobial lipids and their current status and challenges, and to present a detailed discussion of ongoing research efforts to develop nanotechnology formulations of fatty acids and monoglycerides that enable superior *in vitro* and *in vivo* performance. Examples of nano-emulsions, liposomes, solid lipid nanoparticles, and controlled release hydrogels are presented in order to highlight the potential that lies ahead for fatty acids and monoglycerides as next-generation antibacterial solutions. Possible application routes and future directions in research and development are also discussed.

## 1. Introduction

Antibiotic resistance is one of the most serious public health issues in the world, sparked in part by the overuse of antibiotics in medicine and agriculture [1]. In the face of multidrug-resistant bacteria, many antibiotics are losing effectiveness, and there is growing recognition that a post-antibiotic era is approaching [2]. Antibiotics are a key sector of the pharmaceutical industry, as evidenced by high annual expenditures of up to US $10.7 billion in the United States alone [3,4]. The majority of the expenditure comes from outpatient drug prescriptions [4]. The widespread prescription of antibiotics, especially in the outpatient setting, inevitably results in the rise of multidrug-resistant bacterial strains. In recent years, there has been growing recognition of the rise in multidrug-resistance among various bacterial strains as a major public health crisis [5]. Antibiotic-resistant bacterial strains have been estimated to affect 2 million patients annually in the European Union alone [6]. Meanwhile, in the United States, antibiotic-resistance costs more than US $20 billion per year as well as an additional one to two weeks of inpatient care per patient, which strains the existing medical infrastructure [6].

The antibiotic-resistance problem is further aggravated by the fact that new antibiotic drug development has lagged behind the evolution of antibiotic-resistant bacterial strains. From the 1960s up through 2011, merely four new classes of antibiotics were successfully developed and marketed [6]. A 2013 study revealed that only four large pharmaceutical companies have active R & D programs to develop new antibiotics, as compared to 20 companies in the 1980s [3]. Aside from the scientific difficulty in developing new antibiotics, the lack of new antibiotics has been attributed to a perceived lack of potential profit due to competition from low cost, off-patent generic drugs and the short-course nature of antibiotic treatment. Historically unfavorable regulations and policies by government agencies such as the United States Food and Drug Administration (FDA) have also been cited as a factor which discourages the development of new antibiotics [7]. There are increasing demands for narrow-spectrum antibiotics with focused activity against specific bacteria in order to mitigate the chance for antibiotic-resistant bacterial strains to emerge [8]. Broad-spectrum antibacterials with targets that have very high barriers to generating resistance mutations are also in great demand.

Recently, there has been serious attention directed to this issue because the number of drug-resistant bacteria, or so-called super bacteria, continues to rise unabated [9,10]. There is even growing discussion about the end of the antibiotic era altogether. In order to encourage the development of new antibiotics, the FDA has created a new product category called the Qualified Infectious Disease Product (QIDP) [11]. Drugs in this category have a special designation from the FDA, which shortens the approval review process and increases the time (an additional 5 years) for exclusive marketing. These benefits are important because it means that new antibiotics can reach patients more quickly, and there is more economic motivation for pharmaceutical companies to develop new antibiotics. In turn, there has been a positive rise in the number of new antibiotics in the past two years [12]. However, nearly all of these newly approved antibiotics are in fact derivatives of existing antibiotics and share overlapping mechanisms of action. Hence, the problem of drug-resistance quickly emerging is not avoided but rather prolonged, and there is a need for antibiotics against novel bacterial targets, especially those with very high barriers to mutation.

In this regard, membrane-active antibacterial agents hold significant promise [13]. Importantly, the development of resistant bacterial strains against membrane-active compounds has low frequency because there is a high barrier to mutation of the bacterial cell envelope [14]. Membrane-active peptides emerged as an attractive contender and can have potent antibacterial activity [15]. There are many academic studies on membrane-active antibacterial peptides and a few candidates have even reached clinical trials (with at least one in Phase III trials) in the late 90s [16,17]. However, a particularly stringent FDA approval process at the time led to the lack of approval for peptide candidates of that era, and there was a concomitant push in the biotechnology industry at large to move beyond peptide therapeutics [18]. In addition, there are common technical issues with most membrane-active peptides, including weak performance in physiological salt conditions [19], cationic character often renders them toxic to human cells [20], and typical dependence on amino acid secondary structure which is sensitive to environmental conditions. Moreover, membrane-active peptides can be costly to produce [17]. 

At the same time, there is already a documented solution—naturally abundant and low cost free fatty acids and monoglycerides (so-called antimicrobial lipids) with broad-spectrum antibacterial activity—which has long been known, yet was cast aside in favor of small molecule antibiotics for the past few decades [21]. With the current challenges in antibiotic drug development, antimicrobial lipids deserve renewed attention and represent potential solutions to the problem of drug-resistant bacteria. The potential of free fatty acids for biotechnology applications has been highlighted in at least two reviews in the past six years [22,23]. However, arguably the greatest potential for antimicrobial lipids, including free fatty acids, lies in nanotechnology formulations which take advantage of the potent antibacterial properties of these compounds while improving their pharmacological properties and providing superior delivery vehicles. Indeed, these efforts fall under the concept of nanoarchitectonics, an emerging set of design guidelines to incorporate functional molecules into application-oriented nanostructures [24,25,26,27]. In recent years, there have been extensive efforts to achieve the goal of establishing nanotechnology formulations for antimicrobial lipids, yet the collection of research efforts towards this goal has not been summarized.

The aim of this review is to introduce antimicrobial lipids as a class of potent antibacterial agents and to highlight emerging nanotechnology formulations of fatty acids and monoglycerides. An overview of antimicrobial lipids is first provided, followed by a detailed description of formulation strategies based on nano-emulsions, liposomes, solid lipid nanoparticles, and controlled release hydrogels. Finally, the application potential of the nanotechnology formulations is discussed in the context of developing effective solutions to drug-resistant bacteria. 

## 2. Overview of Antimicrobial Lipids

Antimicrobial lipids were first recognized as a component of the human body’s innate immune system. Modern interest in innate antibacterial compounds started with the discovery of the antibacterial enzyme lysozyme in the early 1900s [28]. Since then, the therapeutic potential of additional antibacterial compounds found within the innate immune system, including antimicrobial lipids, has been validated. On the human skin surface, there are two main types of innate host molecules that contribute to antibacterial activity, namely, antimicrobial peptides and antimicrobial lipids [29]. Recent research has revealed that antimicrobial peptides are produced by different classes of human skin cells and human skin prokaryotic microbiota as the immune system’s first line of defense against bacterial infection. As therapeutics, however, antimicrobial peptides are costly to produce in sufficient quantities [28] and have other technical drawbacks as mentioned in the previous section. On the other hand, antimicrobial lipids are abundant in prodigious quantities and exhibit broad-spectrum and strong antimicrobial activity on the skin surface [30].

### 2.1. Brief History

The earliest reports of antimicrobial lipids originated in the late 19th century when Koch observed that free fatty acids inhibit the growth of *Bacillus anthracis* [21]. This finding represented a significant advance in scientific understanding behind the historical role of soaps—alkali salts of fatty acids—as disinfectants and cleaning agents. In the ensuing decades, numerous studies demonstrated that fatty acids inhibit or kill a wide spectrum of pathogens, leading to speculation about possible therapeutic applications [31]. In the 1930s and 1940s, Burtenshaw and contemporaries identified that antimicrobial fatty acids and other lipids are found on human skin and act as natural disinfectants to regulate the skin microbiome [32]. However, the exploration of antimicrobial lipids was tempered by the widespread clinical use of penicillin beginning in the late 1940s, which ushered in the modern era of antibiotics and led to dramatic improvements in human healthcare [33]. 

### 2.2. Classes of Antimicrobial Lipids

Free fatty acids are a widely studied type of antimicrobial lipid, and are composed of a carboxylic acid group and a saturated or unsaturated carbon chain. They function as mild surfactants which perturb bacterial cell membranes [34], causing either bacteriostatic or bactericidal effects [22]. Partial solubilization of the bacterial cell membrane can impair metabolic regulation and/or cellular energy production, which leads to inhibition of bacterial growth. More extensive solubilization, or membrane lysis, can trigger cell death, which often occurs on the time scale of minutes. As such, there are multiple ways by which fatty acids affect bacterial cell membranes; as a result, there is a high barrier to bacterial strains developing resistance mutations [35]. By comparison, antibiotics typically inhibit enzymes involved in specific parts of the bacterial life cycle, thereby increasing the likelihood of resistant strains emerging.

**Figure 1:**
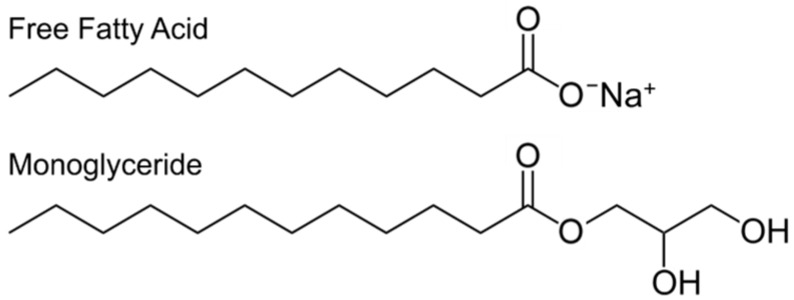
Chemical structures of a representative free fatty acid and monoglyercide.

A wide range of antimicrobial lipids have been discovered or synthesized, and possess a spectrum of antibacterial efficacies and targets. The main approach to classifying antimicrobial lipids has been structure-activity relationship series wherein *in vitro* studies focus on measuring the inhibition of bacterial growth in order to determine the effect of permutations, such as hydrocarbon chain length or the numbers of degrees of unsaturation, within a family of antimicrobial lipids. Kabara and colleagues conducted pioneering studies on the antibacterial properties of medium-chain saturated fatty acids [36,37,38]. Based on detailed studies of saturated fatty acids with chain lengths between 6 and 18 carbons, lauric acid was identified as having the most potent inhibitory activity against Gram-positive bacteria [39]. The 1-monoglyceride derivative of lauric acid is called glycerol monolaurate and was discovered to have a lower minimum inhibitory concentration than lauric acid, albeit against a narrower range of bacteria [40]. Both lauric acid (LA) and glycerol monolaurate (GML) are Generally Recognized As Safe (GRAS) by the United States Food and Drug Administration as food additives [41]. The chemical structures of LA and GML are presented in Figure 1, and highlight the two main classes of antimicrobial lipids that are widely studied: free fatty acids and monoglycerides.

### 2.3. Activity Spectrum

Antimicrobial lipids have demonstrated effectiveness against various drug-resistant bacteria, including methicillin-resistant *Staphylococcus aureus* (MRSA) [29]. Considering the challenges faced by traditional antibiotics, developing therapeutic strategies based on natural compounds which are part of the human innate immune system arsenal is highly advantageous. Antimicrobial lipids are particularly attractive because of their wide abundance in nature, perceived acceptance (several antimicrobial lipids are considered as GRAS by the US FDA), and bactericidal properties against different types of microorganisms including algae, bacteria, fungi, protozoa, and virus [22,36,39]. Owing to a unique mechanism of action, antimicrobial lipids are less prone to the evolution of drug-resistant bacterial strains, as compared to traditional antibiotics. Indeed, antimicrobial lipids interfere with bacterial cell membranes, and can cause cell lysis or a range of indirect effects hindering cell metabolism [22]. As mentioned above, structure-function relationships have shown that optimal bactericidal activity is obtained with saturated antimicrobial lipids with 10- or 12-carbon long fatty acids such as lauric acid, which has successfully exhibited antibacterial activity against a wide range of clinically relevant pathogens, including *S. aureus* and *P. acnes* [22,42]. Antimicrobial lipids with similar chain lengths (10 to 14 carbons) also show potent antimicrobial activity [37]. Other unsaturated fatty acids such as linoleic acid and oleic acid have shown strong antibacterial activity too [43]. Interestingly, in many cases, the activity of a particular compound will overlap but not be identical to that of other antimicrobial lipids [22]. These findings motivate a more detailed understanding of the corresponding mechanisms, yet such information has proven largely elusive.

**Figure 2:**
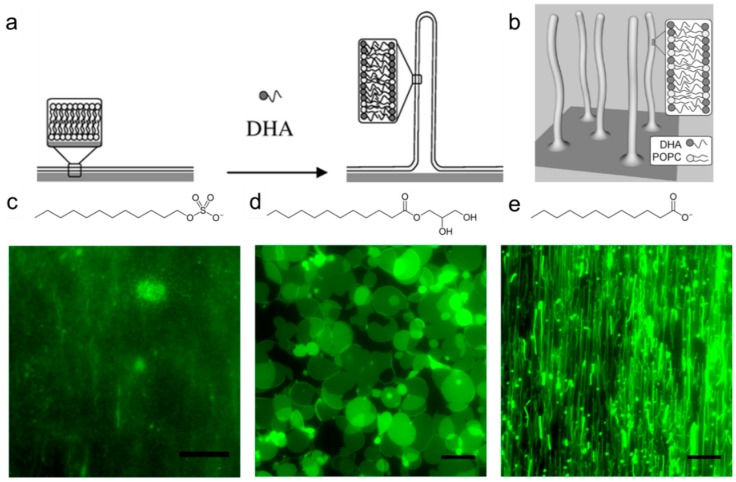
(**a**) Treatment of supported lipid bilayers with docosahexaenoic acid (DHA) induces three-dimensional membrane morphological changes; (**b**) Schematic of DHA-induced, elongated lipid structures on top of supported lipid bilayer. Top-down view of changes in the morphology of supported lipid bilayers, including (**c**) membrane solubilization (SDS treatment); (**d**) membrane budding (GML treatment); and (**e**) membrane fibrillation (LA treatment). The scale bars in all micrographs are 20 μm. Reproduced with permission from Refs. [46,47].

The main focus of structure-activity relationships of fatty acids and monogylcerides has been concentration-dependent measurement of bacterial growth inhibition. The specific interaction between antimicrobial lipids and bacterial cell membranes remains to be understood. Indeed, the lipids present a spectrum of mechanistic behaviors (e.g., permeabilization, lysis, *etc.*), with the specific details depending on the chemical structure and concentration of the lipid [44]. In a few cases, transmission electron microscopy has been utilized in order to analyze fixed specimens of bacteria after treatment with high (5–10 mM) concentrations of certain fatty acids and monoglycerides [45]. However, there have been very few direct investigations of the membrane destabilization process caused by antimicrobial lipids. As presented in Figure 2, recent studies employing model membrane systems called supported lipid bilayers have revealed that free fatty acids and monoglycerides can induce different kinds of morphological changes in the membrane [46,47]. A physicochemical explanation based on the lipid charge and critical micelle concentration was invoked in order to explain the relationship between measured antibacterial activity and fundamental interactions with lipid bilayers [47].

### 2.4. Formulation Challenges

Despite the promising antibacterial features of fatty acids and monoglycerides, there are technical challenges which hinder the *in vivo* performance of compounds in the free form. One drawback that needs to be overcome is the poor solubility of antimicrobial lipids in aqueous buffer solutions [48]. While antimicrobial lipids can be dissolved in solutions containing dimethyl sulfoxide (DMSO), DMSO is a skin irritant and toxic [49]. Other important factors to overcome are the sensitivity to lipid concentration (e.g., critical micelle concentration) and environmental factors (e.g., divalent cations). For these reasons, there has been strong motivation to develop improved formulations that overcome these challenges. In the following section, we describe nanotechnology solutions such as nano-emulsions, liposomes, solid lipid nanoparticles, and controlled release hydrogels which offer significant potential to serve as carriers that not only deliver high concentrations of antimicrobial lipids, but also improve the therapeutic properties of antimicrobial lipids.

## 3. Nanotechnology Strategies

### 3.1. Emulsions

**Figure 3:**
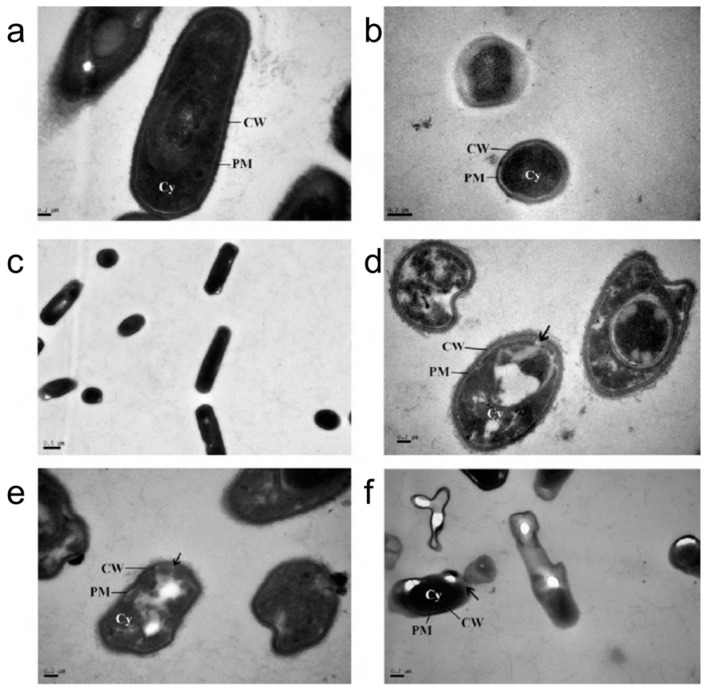
Transmission electron microscopy micrographs of untreated (**a**) *E. coli*; (**b**) *S. aureus*; and (**c**) *B. subtilis* cells. Corresponding TEM mircrographs of microemulsion-treated (**d**) *E. coli*; (**e**) *S. aureus*; and (**f**) *B. subtilis* cells. Reproduced with permission from Reference [57].

Aqueous emulsions composed of immiscible oil droplets in water are commonly prepared with surfactants acting as emulsifiers to reduce tension between the oil and water phases and increase emulsion stability. They can vary in size from the nanoscale to microscale. Owing to attractive properties such as high stability and low viscosity, emulsions are regarded as potential drug delivery systems and are also widely studied for cosmetic, dermatology, food, and paint coating applications. Non-ionic surfactants are commonly chosen because they are less sensitive to environmental conditions, such as the solution pH and ionic strength, and enhance the permeability of cell membranes. Short and medium chain alcohols and related derivatives are also useful surfactants for preparing emulsions, and can display antibacterial activity in the emulsion state. Table 1 summarizes the existing studies reporting antimicrobial effects of emulsions. Al-Adham *et al.* first demonstrated that emulsions show promise as antibacterial agents, with highly effective killing of several different types of bacteria, which was observed with rapid losses of bacterial cell viability upon treatment, supposed to have arisen from membrane-active damage [50]. An example of bacterial cell membrane damage caused by emulsions is presented in Figure 3.

Expanding this research, there has been extensive research focused on exploring monoglycerides as surfactants for preparing emulsions. Considering that monoglycerides are non-ionic surfactants, this choice is not surprising, but it is nonetheless interesting that all research on antibacterial emulsions has strictly focused on monoglycerides without any reported studies on free fatty acids. Thormar *et al.* demonstrated that, among different tested monoglycerides, monocaprin was the most active in killing *C. jejuni*, which is the leading cause of food-borne bacterial infections, and highly diluted monocaprin emulsions retained the ability to cause a >6- to 7-log_10_ reduction in the viable bacterial count within 1 min [51]. However, other research has indicated that the diluted monocaprin emulsions exhibit significant changes in structure and eventually lost all antimicrobial activity. As an alternative solution with greater stability, glycerol monolaurate (GML) emulsions have proven superior and are the most widely studied. GML emulsions were shown to have higher antimicrobial activity than free GML against *B. subtilis* and *E. coli* [52,53,55], and also appeared to have stronger antibacterial effects than the approved antibiotic ceftazidime [56]. GML emulsions can cause complete loss of viability of bacterial cells in 1 min, while 10-fold diluted GML emulsions cause complete loss of viability of bacterial cells within 10 min [54,57]. Acting as solubility enhancers, some salts, e.g., sodium lactate and sodium benzoate, were found to enhance the antimicrobial effects of emulsions in optimized preparations [53,56,57]. Further research indicated that the antimicrobial effects are related to the interaction between the emulsions and bacterial membranes [54,57], after which there is a release of nucleic acid material from inside the bacterial cells as a result of membrane disruption and dysfunction.

Most tested species of both Gram-positive and Gram-negative bacteria were shown to be susceptible to GML emulsions. Fu *et al.* found that the growth of Gram-positive *B. subtilis* was inhibited by GML emulsions more strongly than free GML, while the growth of Gram-negative *E. coli* was inhibited by GML emulsions and free GML to similar extents [55]. Petra *et al.* systematically evaluated the effects of monoglyceride emulsions *versus* free monoglycerides on a panel of Gram- positive and Gram-negative bacteria and determined that Gram-negative strains were more strongly inhibited by monoglyceride emulsions over free monoglycerides, while the opposite trend was observed with Gram-positive bacteria [44]. They also tested different monoglyceride emulsions and found that, among the different monoglycerides, GML emulsions had the strongest antibacterial activity against both bacterial types. Despite the promising antibacterial activity, emulsions can also exhibit toxicity to human cells, and it has been reported that 10 mg/L of GML emulsions kill 40%–60% of treated human cells [58]. For this reason, emulsions appear to be best suited to applications such as food preservatives and highlight the challenges of developing therapeutically viable formulations for fatty acids and monoglycerides.

### 3.2. Liposomes

The original motivation behind liposomal drug delivery (e.g., liposomal doxorubicin [59]) was greater targeting specificity and reduced toxicity of therapeutic agents. Successful liposome delivery systems have been reported for topical and systemic administration routes. In many cases, liposomes have been developed as rigid objects to target infections of the mononuclear phagocyte system and require phagocytic uptake. However, rigid liposomes with zwitterionic character have poor efficacy against extracellular bacteria. To address this challenge, fusogenic liposome preparations with fluid lipid bilayers and negative membrane surface charge have been developed [60]. Fusogenic liposomes that encapsulate traditional antibiotics exhibit strong antibacterial activity, even with only sub-MIC (minimum inhibitory concentration) concentrations of encapsulated antibiotic [61]. The fusogenic liposomes have been shown to fuse with bacterial cell membranes [62], offering a pathway to rescue the antibacterial properties of antibiotics against bacterial strains that have become resistant to the free form of the said antibiotic due to, e.g., decreased membrane permeability or highly functional bacterial cell membrane transporters [63]. Historically, Gram-positive bacteria have been more susceptible to antibiotics, as compared to Gram-negative bacteria, which possess an additional outer membrane. As a delivery vehicle, fusogenic liposomes have a competitive advantage because they not only support targeted delivery, but also enable high uptake of antibacterial agent by bacteria.

Importantly, liposomal delivery vehicles can significantly reduce host cell toxicity and other deleterious side effects of free fatty acids. As presented in this section, there is strong evidence supporting the development of liposomal free fatty acid formulations to reduce bacterial loads, which has been reported in *in vitro* and *in vivo* studies, including both topical and systemic administration routes. Small liposomes loaded with fatty acids encapsulated in the liposomal bilayer can fuse with bacterial cell membranes, leading to release of high local concentrations of fatty acids into the bacterial cell membrane, which results in gross morphological changes and enhanced membrane permeability that causes bacterial cell death [64]. Table 2 presents a summary of existing studies which have utilized liposomal free fatty acids to treat bacterial infections.

**Figure 4:**
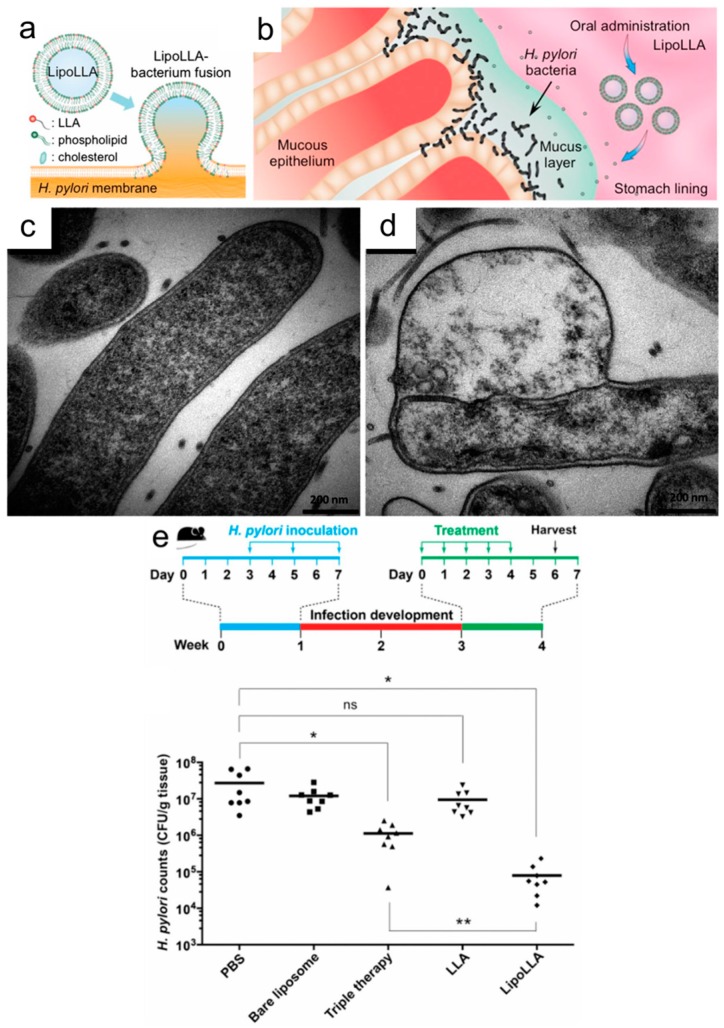
(**a**) Fusion of fatty acid-loaded liposomes with bacterial cell membranes; (**b**) liposomal delivery to *H. pylori*-infected stomach lining; TEM micrographs of (**c**) control and (**d**) LipoLLA-treated *H. pylori* bacteria; (**e**) bacterial load of *H. pylori* in mouse stomach after treatment with different therapeutic agents. * *p* < 0.05, ** *p* < 0.001. Reproduced with permission from Refs. [64,66].

Yang *et al.* first demonstrated that lauric acid-loaded liposomes (LipoLA) can successfully target with a fusion mechanism involving the bacterial cell membrane and completely kill previously resistant strains of *P. acnes* at 51 µg/mL loaded LA concentration [49]. By contrast, the MIC of free LA is around 80 µg/mL. In addition, the antibacterial activity of LipoLA was dependent on the loaded amount of LA per liposome in the formulation with optimal activity achieved at 51% mole fraction [49]. To further explore the therapeutic efficacy of LipoLA, Pornpattananangkul *et al.* evaluated both the *in vitro* and *in vivo* antibacterial activities. *In vitro*, the morphological changes caused by the fusion of LipoLA with bacterial cell membranes and, *in vivo*, the complete killing against *P. acnes*, achieved through intradermal injection and topical administration at 8 mg/mL and 2 mg/mL of LipoLA, respectively, confirmed the potency of LipoLA. Additional toxicity tests of LipoLA on mouse skin showed no irritation, which supports its therapeutic potential against *P. acnes* infection [65]. Meanwhile, there has also been increasing attention to exploring the antibacterial activity of linolenic acid-loaded liposomes (LipoLLA) against *H. pylori* infection, which is related to a range of gastrointestinal diseases including gastric cancer. Obonyo *et al.* identified that LipoLLA has potent antibacterial activity against *H. pylori* in both spiral and coccoid forms, including a diverse range of antibiotic-resistant strains. As presented in Figure 4, distorted morphologies of *H. pylori* are observed with TEM after treatment with LipoLLA, and there is also a sharp decrease in the minimum bactericidal concentration (MBC) values for LipoLLA *versus* free LLA.

Importantly, LipoLLA has a higher barrier for inducing the mutation of drug-resistant strains even after treatment with equivalent LLA concentrations for 10 days, while *H. pylori* eventually acquired resistance mutations to free LLA after only 3 days of treatment [68]. Thamphiwatana *et al.* also explored the therapeutic efficacy of LipoLLA against *H. pylori* infections in an *in vivo* mouse model. It was demonstrated that LipoLLA has superior antibacterial activity against *H. pylori* over free LLA and conventional triple-therapy antibiotics based on measurements of bacterial burden in the mouse stomach after treatment [66]. Further, histopathological analysis of LipoLLA distribution in the mouse stomach showed that LipoLLA was able to penetrate the mucus layer of the mouse stomach and remained in the layer for at least 24 h. LipoLLA also reduced levels of proinflammatory cytokines arising from *H. pylori* infection in the stomach layer without irritation or causing toxicity against tested mouse stomach cells. In order to achieve a greater mechanistic understanding of the fusion process, Jung *et al.* performed detailed characterization studies probing the interaction between LipoLLA and related analogues against *H. pylori* through *in vitro* experiments [64]. The antibacterial activity of liposomal formulations of three different fatty acids in the C18 series, liposomal stearic acid (LSA), liposomal oleic acid (LOA) and LLA, were tested, and it was concluded that LipoLLA had the most potent anti-*H. pylori* activity, with complete killing within 5 min. Moreover, further investigation showed that both LipoLLA and oleic acid-loaded liposome (LipoOA) increased the permeability of the *H. pylori* outer membrane. Interestingly, the permeability of the plasma membrane of cells treated with LipoLLA showed a much greater increase than those treated with LipoOA, as visualized by transmission electron microscopy (TEM) micrographs of *H. pylori* bacteria (*cf.* Figure 4) [64]. Additionally, Huang *et al.* showed that LipoOA is able to treat methicillin-resistant *S. aureus* (MRSA) infection with a 12-fold increase in *in vitro* antibacterial efficacy compared to that of free OA [67]. In the study, intradermally administered LipoOA was demonstrated to have excellent therapeutic potential for treatment of *S. aureus* infections on mouse skin without skin irritation [67]. Based on the aforementioned studies, it is apparent that antimicrobial lipids can target a wide range of bacteria, including at least some drug-resistant strains, and perform well in *in vivo* settings, including topical and systemic administration routes. It also appears that antimicrobial lipids exhibit some degree of selectivity depending on the bacterial cell membrane properties [69].

Interestingly, to date, all liposomal studies have strictly focused on free fatty acids. Considering the wide industrial utilization of monoglycerides, there is significant potential for exploring the development of liposomal formulations for monoglycerides. As described in the previous section, there has been continual exploration of nano-emulsions to host monoglycerides [52,55,57,70,71], yet the critical issue of cytotoxicity remains, and emulsions are also sensitive to dilution and other technical matters for therapeutic applications. Furthermore, monoglycerides are non-ionic surfactants that are less sensitive to environmental conditions than anionic surfactants such as fatty acids. It remains to be investigated whether liposome encapsulation can also improve the therapeutic properties of monoglycerides, and there is a lot of potential for continuing to study liposomal formulations of antimicrobial lipids.

### 3.3. Solid Lipid Nanoparticles

**Figure 5:**
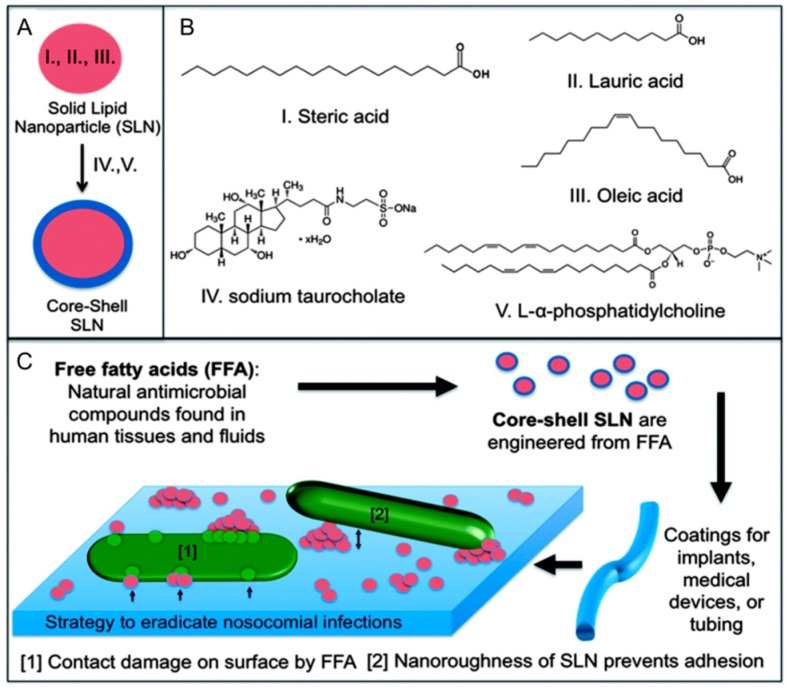
(**A**) Core-shell solid lipid nanoparticles are prepared which contain antimicrobial free fatty acids; (**B**) Lauric acid and oleic acid are antimicrobial lipids which are encapsulated in the inner core; (**C**) Deposited SLNs on the tube surface inhibit bacterial adhesion and damage bacteria. Reproduced with permission from Reference [72].

Antibiotic-resistant bacterial infections caused by invasive medical devices (e.g., endotracheal tubes) represent a major challenge that requires new treatment strategies. In particular, there is demand for designing antibacterial surface coatings that prevent bacterial infections without relying on antibiotic solutions. To address this need, Taylor *et al.* developed a solid lipid nanoparticle (SLN) formulation that consisted of lauric acid, stearic acid, and oleic acid in the inner core which was surrounded by phosphatidylcholine lipid and sodium taurocholate (Figure 5) [72]. The aim of the design was to kill adhered bacteria based on the membrane-disruptive activities of the encapsulated lauric acid and oleic acid. The SLNs were deposited on the surface of endotracheal tubing, and the effect on *Pseudomonas aeruginosa* morphology and viability was evaluated. It was determined that SLNs reduced bacterial cell adhesion by 99%, destabilized the structure of the few adhered cells, and inhibited bacterial growth. SLNs loaded with retinoic acid and lauric acid have also been developed and inhibited the growth of *S. epidermidis*, *P. acnes*, and *S. aureus* [73].

### 3.4. Hydrogel

Hydrogels composed of hydrophilic polymers with three-dimensional cross-linked structures are biocompatible materials that contain a large fraction of water, and are promising drug delivery vehicles with nanoscale mesh networks [74]. Importantly, they have also been investigated for microbicidal applications based on the encapsulation and controlled release of monoglycerides. Table 3 provides a summary of existing studies that have utilized hydrogel vehicles for the encapsulation of free fatty acids and monoglycerides. Kristmundsdóttir *et al.* originally explored how to develop hydrogel formulations containing a topical microbicide for preventing sexually transmitted viruses, e.g., herpes simplex virus type 1 (HSV-1) and human immunodeficiency virus (HIV) [75]. Virucidal activity was tested with a wide range of medium- and long-chain fatty acids and their monoglyceride derivatives against HSV-1, and monocaprin, the 1-monoglyceride of capric acid, was selected for further development in a hydrogel formulation based on its demonstration of the most potent antiviral activity at 20 mM free concentration. Moreover, an optimal hydrogel formulation containing monocaprin along with other additives was prepared. The optimized hydrogel formulation had significant antiviral activity against HSV-1 with greater than 10^5^-fold inactivation within 1 min in *in vitro* experiments. In addition, it was less cytotoxic to a human cell line than a commercial spermicidal product tested in parallel. On the basis of the developed hydrogel formulation containing monocaprin, the gel was further investigated against herpes simplex virus type 2 (HSV-2), HIV-1, *Chlamydia trachomatis*, and *Neisseria gonorrhoeae*, and high inactivation efficacy of the gels was confirmed against all tested pathogens without irritation of rabbit vaginal mucosa [76]. This was the first report of antibacterial activity of monoglycerides in a hydrogel formulation.

Meanwhile, additional studies have focused on characterization of the physicochemical properties of hydrogel formulations containing monocaprin. Thorgeirsdottir *et al.* showed enhanced stability of the monoglyceride in the hydrogel formation in the presence of Carbomer 974P, while the surfactant, polysorbate 20, decreased HSV-1 inactivation [77]. Furthermore, Kristmundsdóttir *et al.* investigated the effect of buffer type on the antiviral activity and physicochemical properties of hydrogel containing monocaprin in order to prevent infection in acidic environments such as the vaginal region [78]. Two different gel formulations were prepared by using gelling agents, sodium carboxymethyl cellulose (NaCMC) and carbomer, and tested with malate or citrate/lactate buffers. The presence of the buffers led to a decrease in hydrogel viscosity, while the antiviral activity of monocaprin in the gels remained unchanged. In another study, Thorgeirsdóttir *et al.* scrutinized the effect of surfactant addition, the presence of monocaprin, and the solution pH on the rheological and structural properties of the hydrogel formulation [67]. The addition of non-ionic surfactants and monocaprin significantly impacted the rheological features of the formulation at pH 4, while the effect was moderate in the higher pH range of 5–7 [79]. All these characterization studies point to the detailed consideration of possible hydrogel formulations for controlled release in the vaginal region, although other applications remain to be explored beyond sexually transmitted diseases.

To explore the therapeutic efficacy of the hydrogel formulation including monocaprin, Neyts *et al.* performed *in vivo* antiviral tests against HIV-2 infection in mice [80]. The gel efficiently prevented intravaginal and intracutaneous HIV-2 infection in mice at 20 mM monocaprin without irritation or toxicity against the mice skin, supporting that the hydrogel formulation containing monocaprin is promising for vaginal microbicidal applications. Skulason *et al.* reported the findings of a clinical trial which evaluated the efficacy of a hydrogel formulation that included monocaprin and low dose doxycycline antibiotic, and was applied for the treatment of *Herpes labialis* [81]. Importantly, the formulation was active against *Herpes labialis* in both the prodromal and vesicular stages, with a significant decrease in healing time and pain in comparison with a placebo hydrogel. While most hydrogel studies have focused on antiviral applications, hydrogel formulations have also demonstrated some utility for antibacterial applications (e.g., against *C. trachomatis* and *N. gonorrhoeae*), and further investigation is warranted, especially for topical skin usage.

## 4. Conclusions

With the growing challenges of multidrug-resistant bacteria, the development of new antibacterial solutions continues to become more urgent. Antimicrobial lipids demonstrate a number of promising features that make them attractive options for antibacterial applications, including wide and renewable abundance, low cost, and a broad spectrum of activity. At the same time, there have been technical challenges associated with their translation into industrial applications, especially as therapeutic options. As discussed herein, nanotechnology formulations are providing new hope for the development of effective antibacterial solutions based on antimicrobial lipids. While nanomedicine is typically associated with complex architectures and sophisticated features, the examples provided in this article suggest that nanomedicine has great potential for even relatively simple designs. The nanotechnology formulations which have been utilized for encapsulating antimicrobial lipids are well-established and can be easily scaled up—attractive features which motivate the further exploration of nanotechnology formulations for antimicrobial lipids.

One of the most surprising findings that we noticed about the ongoing work in this field is how different kinds of nanotechnology formulations have almost exclusively focused on encapsulating either free fatty acids or monoglycerides. Indeed, to our knowledge, essentially all studies on nano-emulsions and controlled release hydrogels have involved monoglycerides. Furthermore, the reported liposome and solid lipid nanoparticle studies have exclusively involved fatty acids. Based on this finding, there is significant opportunity for exploring new combinations of nanotechnology vehicle and antimicrobial lipid. As our mechanistic understanding of antimicrobial lipids continues to improve through detailed biophysical measurements (*cf.* Figure 2), it is becoming increasingly apparent that subtle variations in the structure of antimicrobial lipids can impart significant effects on how the lipids perturb bacterial cell membranes, in turn casting light on the overlapping yet different spectrums of antibacterial activities for various antimicrobial lipids. A deeper understanding of how different classes of antimicrobial lipids function in nanotechnology formulations will help to identify the optimal strategies for translating research prototypes into viable solutions for specific applications.

Based on the work conducted thus far, it appears that emulsion systems will be best suited for food preservatives and other industrial applications where the diluted concentrations of active agents once ingested are below those at which human cell cytotoxicity becomes an issue. The main reason behind this recommendation is that the emulsions sequester high concentrations of antimicrobial lipid which make them potent and broad-spectrum antibacterial agents, yet do not reduce cytotoxicity. On the other hand, liposomes are particularly attractive systems because they enable antibacterial activity at effective concentrations below the CMC value of the encapsulated fatty acid and have minimal human cell cytotoxicity. The demonstrated *in vivo* performance further reinforces the potential of liposomal strategies to take advantage of antimicrobial lipids for therapeutic applications. While hydrogel systems have mainly focused on antiviral applications, they also show strong promise for antibacterial applications as described above (*cf.* Table 3), and one could envision topical applications against bacterial skin infections. Solid lipid nanoparticles are an emerging system that has just begun to be studied, and yet already shows promising results that warrant further exploration. Another important objective should be to compare the performance of different types of nanotechnology formulations in more application-specific contexts. Altogether, significant progress is being made to realize the potential of antimicrobial lipids by employing nanotechnology formulations. Future research and development in this direction holds great potential to help stem the challenges of multidrug-resistant bacteria.

Considering the promise of research strategies in this direction, it is seemingly counterintuitive at first glance that research and development on this topic has proceeded rather gradually. Scientifically, while antimicrobial lipids in the free form can have potent antibacterial activity, their *in vivo* performance in human patients (often in the form of dietary supplements [82,83]) has presented conflicting results about their efficacy in reducing bacterial loads. These challenges may relate to the dependence of antibacterial activity on the effective lipid concentration and corresponding relationship with the critical micelle concentration. Nanotechnology formulations such as liposomes, which preserve antibacterial activity below the CMC value of encapsulated lipids, offer promise to address this challenge, as evidenced by the recent work by Thamphiwatana *et al.* [66] At the same time, there have been international reports that certain antimicrobial lipids, including glycerol monolaurate, can reduce viral loads in HIV-infected patients [84]. It should be stressed that such findings are preliminary and warrant careful scrutiny, but also suggest that further studies are warranted, especially in the United States and Europe, where such studies are generally lacking. Economically, the question turns to how clinical studies on antimicrobial lipids can be motivated considering that the natural compounds are not patentable. Indeed, the lack of intellectual property in the field has been an important factor stymieing product development [23]. From this perspective, nanotechnology formulations for antimicrobial lipids are promising because research achievements could not only improve the therapeutic performance of such compounds, but also lead to patentable inventions that may stimulate further translation of lead candidates from bench to bedside. With these goals in mind, great potential lies ahead for developing nanotechnology formulations for antimicrobial lipids.

## Figures and Tables

**Table 1:** Past studies involving antibacterial properties of monoglyceride emulsions.

Antimicrobial Lipid	Organism	Effects	Reference
Ethyl oleate	*E. coli*	• Bactericidal activity against *Ps. aeruginosa* and *S. aureus*, and induced membrane alterations against *Ps. Aeruginosa*.	[50]
	*Ps. aeruginosa*		
	*S. aureus*		
Monocaprin	*C. jejuni*	• ~1 mM monocaprin emulsions caused a greater than 6- to 7-log_10_ reduction in viable bacterial count of *C. jejuni* within 1 min. • Antimicrobial activity of monocaprin emulsions against *C. coli*, *C. lari*, *Salmonella* spp. and *E. coli*. was demonstrated.	[51]
	*C. coli*		
	*C. lari*		
	*Salmonella* spp.		
	*E. coli*		
Glycerol monolaurate	*B. subtilis*	• Increased antibacterial activity of GML emulsions *versus* free GML against *B. subtilis* and *E. coli*.	[52]
	*E. coli*		
Glycerol monolaurate	*B. subtilis*	• Demonstrated antibacterial effect of emulsions against *B. subtilis*, with improved activity in the presence of sodium lactate salt.	[53]
Glycerol monolaurate	*E. coli*	• Complete loss of viability of *E. coli* or *S. aureus* cells within 1 min caused by highly concentrated emulsions, and slow kinetics observed with 10-times diluted emulsions. • Induced release of nucleic acids due to bacterial membrane damage.	[54]
	*S. aureus*		
Glycerol monolaurate	*B. subtilis*	• GML emulsions are more potent against *B. subtilis* whereas free GML is stronger against *E. coli.*	[55]
	*E. coli*		
Glycerol monolaurate	*S. maltophilia*	• Greater antibacterial activity of GML emulsions against *S. maltophilia versus* the ceftazidime antibiotic, and enhanced activity with sodium benzoate as a hydrotrope.	[56]
	*E. coli*		
Glycerol monolaurate	*B. subtilis*	• Complete loss of viability of *E. coli*, *S. aureus* and *B. subtilis* cells within 1 min caused by GML emulsions. • Emulsions damaged bacterial cell walls.	[57]
	*E. coli*		
	*S. aureus*		
1-monoacylglycerol (1-MAG) of capric (C10:0), undecanoic (C11:0), lauric (C12:0),, myristic (C14:0) acids	*B. cereus*	• Enhanced antimicrobial activities of emulsions against Gram-negative strains *versus* 1-MAGs and opposite trend was observed with Gram-positive stains. • Best antibacterial activity against both bacterial types was observed with 1-MAG C12:0 emulsions. • 10 mg/L concentration was determined to be the limit for moderate toxicity (40%–60% cell survival).	[58]
	*B. subtilis*		
	*E. faecalis*		
	*S. aureus*		
	*M. luteus*		
	*C. freundii*		
	*E. coli*		
	*P. aeruginosa*		
	*S. entérica*		
	*S. marcescens*		

**Table 2:** Past studies involving antibacterial properties of liposomal fatty acids.

Antimicrobial Lipid	Organism	Study Design	Effects	Reference
Linolenic acid, Stearic acid, Oleic acid	*H. pylori*	*In Vitro*	• Demonstrated efficacy of LipoLLA with MBC value of 200 µg/mL.	[65]
			• Significant effect of LipoLLA on increasing outer membrane permeability of *H. pylori*.	
Linolenic acid	*H. pylori*	*In Vitro*	• Effective in killing both spiral and coccoid forms of the bacteria based on membrane disruption.	[66]
			• LipoLLA has higher barrier to development of drug-resistant strains than free LLA.	
Lauric acid	*P. acnes*	*In Vitro*	• MBC value of LipoLA at 51 µg/mL against *P. acnes*.	[50]
			• Established importance of critical molar fraction of free fatty acids in LipoFAs.	
Linolenic acid	*H. pylori*	*In Vivo*	• MBC values for LipoLLA and LLA of 65 µg/mL and 80 µg/mL, respectively.	[67]
			• Significant efficacy of LipoLLA *in vivo* with excellent biocompatibility.	
Lauric acid	*P. acnes*	*In Vivo*	• Effective therapeutic efficacy of 2 mg/mL LipoLA in topical formulation.	[68]
			• No irritation of normal mouse skin by LipoLLA.	
Oleic acid	*S. aureus* (MRSA)	*In Vivo*	• 12-fold increase in *in vitro* efficacy of OA in liposomal formulation *versus* free OA.	[69]
			• High *in vivo* efficacy of LipoOA in treatment of MRSA skin infections.	

**Table 3:** Past studies involving antimicrobial properties of hydrogel encapsulation strategies.

Antimicrobial Agents	Organism	Study Design	Effects	Reference
Caprylic acid, Capric acid, Undecylenic acid, Lauric acid, Myristic acid, Palmitoleic acid, Oleic acid, 1-monoglyceride of each fatty acid	Herpes simplex virus type 1 (HSV-1)	*In Vitro*	• Monocaprin, the 1-monoglyceride of capric acid, had the most potent antiviral activity compounds against HSV-1.	[75]
			• Significant antiviral activity of hydrogel formulation against HSV-1 with > 10^6^-fold inactivation in 1 min.	
			• Reduced cytotoxicity in human cells with monocaprin-containing hydrogel compared to commercial spermicidal product.	
Monocaprin	Herpes simplex virus type 2 (HSV-2), HIV-1, *C. trachomatis*, *N. gonorrhoeae*	*In Vitro*	• Potent inactivation efficacy against HSV-2, HIV-1, *N. gonorrhoeae,* and *C. Trachomatis.*	[76]
			• No toxicity of the monocaprin-containing hydrogel against rabbit vaginal mucosa.	
Monocaprin	Herpes simplex virus (HSV-1)	*In Vitro*	• Enhanced stability of monocaprin in pharmaceutical formulations in the presence of carbomer 974P.	[77]
			• Reduced effect on HSV-1 inactivation with increasing amount of polysorbate 20 surfactant.	
Monocaprin	Herpes simplex virus (HSV-1)	*In Vitro*	• Insignificant effect of buffer on antiviral activity of hydrogel formulation against HSV-1.	[78]
			• Decreased hydrogel viscosity in the presence of buffers.	
Monocaprin	N.A.	*In Vitro*	• Significant effect of solution pH on structural and rheological properties of hydrogels.	[79]
Monocaprin	Herpes simplex virus (HSV-2)	*In Vivo*	• Potent antiviral activity of hydrogel formulation against intravaginal and intracutaneous HSV-2 infection in mice without skin irritation.	[80]
Monocaprin Doxycyclin	*Herpes labialis*	*In Vivo*	• Effective clinical treatment of hydrogel formulation containing monocaprin and doxycyclin for treatment of *Herpes labialis*.	[81]
			• Significant decrease in healing time and pain *versus* free monocaprin and placebo hydrogel.	
